# HIV Testing Among Transgender Women and Men — 27 States and Guam, 2014–2015

**DOI:** 10.15585/mmwr.mm6633a3

**Published:** 2017-08-25

**Authors:** Marc A. Pitasi, Emeka Oraka, Hollie Clark, Machell Town, Elizabeth A. DiNenno

**Affiliations:** ^1^Division of HIV/AIDS Prevention, National Center for HIV, Viral Hepatitis, STD and TB Prevention, CDC; ^2^Division of Population Health, National Center for Chronic Disease Prevention and Health Promotion, CDC.

Transgender persons are at high risk for human immunodeficiency virus (HIV) infection; in a recent analysis of the results of over nine million CDC funded HIV tests, transgender women* had the highest percentage of confirmed positive results (2.7%) of any gender category (*1*). Transgender men,^†^ particularly those who have sex with cisgender^§^ men, are also at high risk for infection (*2*). HIV testing is critical for detecting and treating persons who are infected and delivering preventive services to those who are uninfected. CDC recommends that persons at high risk for HIV infection be screened for HIV at least annually, although transgender persons are not specified in the current recommendations. CDC analyzed data from the Behavioral Risk Factor Surveillance System (BRFSS) to describe HIV testing among transgender women and men and two cisgender comparison groups in 27 states and Guam. After adjusting for demographic characteristics, transgender women and men had a lower prevalence of ever testing and past year testing for HIV (35.6% and 31.6% ever, and 10.0% and 10.2% past year, respectively) compared with cisgender gay and bisexual men (61.8% ever and 21.6% past year) and instead reported testing at levels comparable to cisgender heterosexual men and women (35.2% ever, and 8.6% past year). This finding suggests that transgender women and men might not be sufficiently reached by current HIV testing measures. Tailoring HIV testing activities to overcome the unique barriers faced by transgender women and men might increase rates of testing among these populations.

BRFSS is an annual, state-based, random-digit–dialed cellular and landline telephone survey of the noninstitutionalized U.S. adult population.^¶^ Gender identity was uniformly assessed in an optional module used by 20 jurisdictions** in 2014 and 22 jurisdictions^††^ in 2015. Fourteen jurisdictions participated in the module during both years, six participated only in 2014, and eight participated only in 2015, for a total of 28. Jurisdiction-specific response rates ranged from 33.0% to 59.2%^§§^ and 34.4% to 57.6%^¶¶^ in 2014 and 2015, respectively. Transgender respondents were defined as those who answered affirmative to the question if they considered themselves to be transgender. Those who answered affirmative were asked to identify as male-to-female (defined as transgender women in this report), female-to-male (defined as transgender men in this report), or gender nonconforming. Because of small sample size, responses from gender nonconforming persons (n = 272) were not included in this analysis.

Pooled data collected in 2014 and 2015 were used to compare demographic characteristics and HIV testing among transgender and cisgender respondents. Cisgender men who reported sexual orientations of gay or bisexual represent a group at high risk for HIV infection (*3*). Cisgender men and women who reported an orientation of straight (hereafter referred to as cisgender heterosexual men and women) represent a group at lower risk for infection (*4*). The proportion of respondents who reported ever and past year HIV testing was calculated, and unadjusted prevalence ratios and 95% confidence intervals were estimated to identify characteristics associated with ever testing among transgender women and men. Multivariable logistic regression models compared self-reported prevalence of ever and past year testing among transgender women and men with cisgender gay and bisexual men while adjusting for characteristics associated with testing in univariate models (p<0.10). All estimates were weighted to account for the complex multistage sampling design; because only 14 of 28 jurisdictions participated in the optional module during both years of data collection, weights for these 14 jurisdictions were averaged across the 2-year period to account for varying levels of participation over time. Estimates with relative standard error ≥30% were not reported.

During 2014–2015, 28 jurisdictions collected data on gender identity, resulting in a total sample of 732 transgender women, 451 transgender men, 3,798 cisgender gay and bisexual men, and 301,524 cisgender heterosexual men and women (Table 1). The unadjusted prevalence of ever testing for HIV was 37.5% among transgender women, 36.6% among transgender men, 66.2% among cisgender gay and bisexual men, and 35.2% among cisgender heterosexual men and women. The unadjusted prevalence of past year testing was 11.7% among transgender women, 12.4% among transgender men, 27.5% among cisgender gay and bisexual men, and 8.6% among cisgender heterosexual men and women.

**TABLE 1:** Selected demographic characteristics and HIV testing behaviors among transgender and cisgender respondents* — Behavioral Risk Factor Surveillance System, 27 states and Guam,^†^ 2014–2015

Characteristic	Transgender women		Transgender men		Cisgender gay and bisexual men^§^		Cisgender heterosexual men and women^¶^	
	No.	%** (95% CI)	No.	%** (95% CI)	No.	%** (95% CI)	No.	%** (95% CI)
Total	732	100 —	451	100 (—)	3,798	100 (—)	301,524	100 (—)
**Race/Ethnicity**								
White, non-Hispanic	527	60.6 (52.8–67.9)	309	49.2 (37.9–60.6)	2,929	67.1 (63.7–70.3)	242,370	71.1 (70.7–71.5)
Black, non-Hispanic	67	13.3 (9.3–18.7)	43	11.4 (6.6–18.9)	233	11.8 (9.7–14.5)	21,166	12.0 (11.7–12.3)
Hispanic or Latino	46	13.2 (8.1–21.0)	48	29.0 (18.6–42.2)	250	12.8 (10.2–15.8)	14,320	11.0 (10.7–11.4)
Other, non-Hispanic	77	12.9 (7.9–20.2)	42	^—††^	338	8.3 (6.9–10.1)	19,890	5.9 (5.7–6.1)
**Age group (yrs)**								
18–24	55	14.2 (9.9–19.8)	30	15.6 (8.7–26.4)	434	21.9 (19.0–25.1)	14,166	11.7 (11.4–12.0)
25–44	146	29.1 (22.3–37.0)	98	45.2 (33.7–57.2)	893	33.4 (30.5–36.5)	60,098	31.5 (31.1–31.9)
45–64	322	40.3 (33.6–47.4)	185	25.0 (18.3–33.1)	1,582	33.9 (31.2–36.7)	122,321	36.2 (35.9–36.6)
≥65	209	16.4 (12.6–21.1)	138	14.2 (10.0–19.8)	889	10.8 (9.5–12.3)	104,939	20.6 (20.4–20.8)
**Education**								
<High school	90	22.0 (15.8–29.9)	72	34.4 (23.5–47.1)	180	10.4 (8.2–12.9)	19,081	12.2 (11.9–12.5)
High school	292	38.5 (32.0–45.4)	169	40.6 (29.9–52.2)	813	25.4 (22.5–28.5)	86,020	29.7 (29.4–30.1)
Some college	205	24.2 (18.6–30.9)	116	15.1 (10.2–21.8)	996	31.5 (28.6–34.5)	82,460	31.1 (30.7–31.5)
College or above	142	15.3 (11.3–20.4)	92	10.0 (6.5–15.0)	1,801	32.8 (30.2–35.5)	113,289	27.0 (26.7–27.3)
**Annual household income**								
<$25,000	240	40.1 (33.0–47.6)	149	30.4 (21.7–40.8)	950	25.5 (22.9–28.3)	64,039	22.3 (21.9–22.6)
$25,000–$49,999	169	20.9 (15.6–27.4)	118	29.4 (19.7–41.4)	886	21.7 (19.2–24.4)	66,938	21.3 (21.0–21.7)
≥$50,000	232	29.5 (23.8–35.9)	120	24.4 (15.0–37.0)	1,604	40.1 (37.2–43.1)	128,546	42.8 (42.4–43.1)
Missing	91	9.6 (6.4–14.0)	64	15.8 (10.0–24.1)	358	12.8 (10.4–15.6)	42,001	13.7 (13.4–13.9)
**Has health insurance**								
Yes	649	80.7 (72.7–86.8)	394	70.6 (57.1–81.2)	3,446	88.2 (86.1–90.1)	280,774	88.8 (88.5–89.1)
No	73	19.3 (13.2–27.4)	54	29.5 (18.8–42.9)	338	11.8 (9.9–13.9)	19,804	11.2 (10.9–11.5)
**Marital status**								
Married or unmarried couple	383	52.2 (45.0–59.2)	219	53.1 (41.8–64.1)	1,302	33.2 (30.5–36.1)	172,305	57.3 (56.9–57.7)
Separated/widowed/ divorced	184	19.7 (15.0–25.5)	138	18.5 (12.4–26.8)	551	10.6 (8.9–12.6)	85,996	20.5 (20.2–20.8)
Never married	161	28.1 (22.3–34.7)	90	28.4 (19.7–39.0)	1,918	56.2 (53.1–59.2)	41,852	22.2 (21.8–22.5)
**Geographic region**								
Northeast	111	21.5 (16.7–27.2)	48	16.3 (10.7–24.0)	904	30.3 (27.8–33.0)	50,129	25.6 (25.3–25.8)
Midwest	309	36.2 (29.9–43.0)	206	34.5 (24.9–45.5)	1,358	29.2 (26.6–31.9)	122,255	33.0 (32.7–33.2)
South	186	37.1 (29.8–45.0)	133	42.4 (31.1–54.6)	907	32.0 (28.7–35.4)	77,703	33.8 (33.5–34.1)
West	114	5.3 (3.9–7.2)	58	6.8 (4.2–10.7)	595	8.5 (7.4–9.8)	49,389	7.7 (7.6–7.8)
**County of residence**								
Metropolitan	484	77.3 (71.3–82.4)	304	80.3 (71.7–86.8)	3,020	87.4 (85.2–89.3)	210,875	81.5 (81.3–81.7)
Nonmetropolitan	236	22.7 (17.6–28.7)	141	19.7 (13.2–28.3)	744	12.6 (10.7–14.8)	88,601	18.5 (18.3–18.7)
**Ever received diagnosis of depressive disorder**								
Yes	149	21.9 (16.8–28.1)	116	22.6 (15.2–32.1)	1,156	30.9 (28.0–34.0)	56,693	18.0 (17.7–18.3)
No	577	78.1 (71.9–83.2)	331	77.4 (67.9–84.8)	2,619	69.1 (66.0–72.1)	243,693	82.0 (81.7–82.3)
**Ever tested for HIV**								
Yes	225	37.5 (30.8–44.6)	137	36.6 (27.0–47.4)	2,506	66.2 (63.3–69.1)	80,241	35.2 (34.8–35.5)
No	499	62.6 (55.4–69.2)	302	63.4 (52.6–73.0)	1,247	33.8 (30.9–36.7)	211,990	64.8 (64.5–65.2)
**Tested for HIV in past 12 months**								
Yes	65	11.7 (7.9–16.9)	29	12.4 (6.8–21.5)	895	27.5 (24.9–30.3)	15,118	8.6 (8.4–8.9)
No	667	88.3 (83.1–92.1)	422	87.6 (78.5–93.2)	2,903	72.5 (69.7–75.1)	286,406	91.4 (91.1–91.6)
**Setting of last HIV test**								
Private doctor/HMO/clinic	136	58.9 (47.4–69.6)	91	61.4 (44.9–75.6)	1,651	65.1 (61.4–68.7)	53,626	69.6 (69.0–70.3)
Hospital (inpatient and ED)	34	14.5 (8.1–24.5)	25	13.5 (7.5–23.1)	222	9.2 (7.4–11.4)	9,498	12.0 (11.6–12.5)
Other^§§^	51	26.7 (17.9–37.8)	20	^—††^	605	25.6 (22.3–29.3)	15,606	18.4 (17.8–18.9)

**Abbreviations:** CI = confidence interval; ED = emergency department; HIV = human immunodeficiency virus; HMO = health maintenance organization.

* Chi-square tests were conducted to assess differences in demographic characteristics and HIV testing behaviors between gender identity categories; all p ≤0.01 (data not shown).

^†^ Data were collected in the following jurisdictions: Colorado (2015), Connecticut (2015), Delaware, Georgia (2015), Guam (2014). Hawaii, Idaho, Illinois (2015), Indiana, Iowa, Kansas, Kentucky (2014), Louisiana (2014), Maryland, Massachusetts (2015), Minnesota, Missouri (2015), Montana (2014), Nevada, New York, Ohio, Pennsylvania, Texas (2015), Vermont (2014), Virginia, West Virginia (2015), Wisconsin, and Wyoming (2014).

^¶^ Includes cisgender men and women who reported a sexual orientation of straight.

** Weighted column percent excludes missing values and responses of don't know, not sure, or not asked unless otherwise noted.

^††^ Estimate suppressed because relative standard error of the estimate was ≥30%.

^§§^ Includes counseling and testing sites, correctional facilities, drug treatment facilities, at home, or somewhere else.

Black transgender women (62.6%) and men (66.9%) had a higher prevalence of ever testing than their white counterparts (33.2% and 30.7%, respectively). Among transgender women, the highest prevalence of ever testing (68.5%) was reported by those who had ever received a diagnosis of a depressive disorder (Table 2). After adjusting for demographic characteristics, transgender women and men had a lower prevalence of ever testing and past year testing for HIV (35.6% and 31.6% ever, and 10.0% and 10.2% past year, respectively) compared with cisgender gay and bisexual men (61.8% ever, and 21.6% past year) and reported testing at levels comparable with those of cisgender heterosexual men and women (35.2% ever, and 8.6% past year) (Table 3).

**TABLE 2:** Prevalence of ever testing for HIV by demographic characteristics among transgender women and men — Behavioral Risk Factor Surveillance System, 27 states and Guam,* 2014–2015

Characteristic	Transgender women		Transgender men		
	% Ever tested^†^ (95% CI)	PR (95% CI)	% Ever tested^†^ (95% CI)	PR (95% CI)	
**Race/Ethnicity**					
White, non-Hispanic	33.2 (25.7–41.6)	Ref	30.7 (21.9–41.2)	Ref	
Black, non-Hispanic	62.6 (45.2–77.3)	1.9 (1.3–2.7)	66.9 (42.8–84.6)	2.2 (1.4–3.4)	
Hispanic or Latino	^—§^	1.0 (0.5–2.0)	^—§^	1.3 (0.7–2.6)	
Other, non-Hispanic	^—§^	0.9 (0.4–1.9)	^—§^	0.5 (0.1–2.2)	
**Age group (yrs)**					
18–24	34.7 (19.9–53.2)	0.6 (0.4–1.1)	53.6 (25.6–79.6)	1.3 (0.6–2.8)	
25–44	54.4 (38.1–69.9)	Ref	40.2 (23.4–59.6)	Ref	
45–64	35.1 (25.8–45.7)	0.6 (0.4–1.0)	36.2 (23.9–50.6)	0.9 (0.5–1.7)	
≥65	14.7 (8.1–25.2)	0.3 (0.1–0.5)	^—§^	0.2 (0.1–0.4)	
**Education**					
<High school	36.4 (21.4–54.6)	1.0 (0.6–1.7)	^—§^	0.9 (0.4–1.9)	
High school	37.6 (27.5–49.0)	Ref	38.0 (23.0–55.8)	Ref	
Some college	39.3 (26.9–53.2)	1.0 (0.7–1.6)	28.4 (17.4–42.7)	0.8 (0.4–1.4)	
College or above	35.1 (22.1–50.9)	0.9 (0.6–1.6)	58.9 (40.9–74.8)	1.6 (0.9–2.7)	
**Annual household income**					
<$25,000	41.8 (30.4–54.1)	1.2 (0.7–2.0)	51.5 (34.4–68.2)	1.6 (0.8–3.0)	
$25,000–$49,999	35.4 (21.8–51.9)	Ref	32.8 (17.3–53.1)	Ref	
≥$50,000	32.1 (21.8–44.5)	0.9 (0.5–1.6)	^—§^	0.7 (0.3–1.7)	
**Has health insurance**					
Yes	36.4 (29.5–43.9)	Ref	39.1 (28.7–50.7)		Ref
No	40.2 (22.3–61.1)	1.1 (0.6–1.9)	^—§^		0.8 (0.4–1.7)
**Marital status**					
Married or unmarried couple	31.3 (22.4–41.8)	Ref	25.0 (15.1–38.5)		Ref
Separated/widowed/divorced	44.0 (30.6–58.4)	1.4 (0.9–2.2)	47.8 (28.2–68.1)		1.9 (1.0–3.7)
Never married	44.5 (32.5–57.2)	1.4 (0.9–2.2)	49.9 (31.6–68.3)		2.0 (1.1–3.7)
**Geographic region**					
Northeast	33.4 (22.3–46.6)	Ref	38.5 (20.9–59.8)		Ref
Midwest	35.7 (25.9–46.8)	1.1 (0.7–1.7)	36.6 (21.1–55.5)		1.0 (0.5–2.0)
South	41.2 (28.3–55.6)	1.2 (0.8–2.0)	35.5 (20.6–53.8)		0.9 (0.5–1.9)
West	39.8 (27.0–54.1)	1.2 (0.7–2.0)	39.9 (20.7–62.8)		1.0 (0.5–2.2)
**County of residence**					
Metropolitan	35.9 (28.5–44.1)	Ref	40.4 (28.6–53.4)		Ref
Nonmetropolitan	42.7 (29.7–56.8)	1.2 (0.8–1.8)	^—§^		0.5 (0.3–1.1)
**Ever received diagnosis of depressive disorder**					
Yes	68.5 (54.7–79.6)	2.4 (1.8–3.3)	40.4 (23.9–59.4)		1.2 (0.7–2.1)
No	28.5 (21.8–36.4)	Ref	34.9 (23.8–47.9)		Ref

**Abbreviations:** CI = confidence interval; HIV = human immunodeficiency virus; PR = prevalence ratio; Ref = reference category.

* Data were collected in the following jurisdictions: Colorado (2015), Connecticut (2015), Delaware, Georgia (2015), Guam (2014), Hawaii, Idaho, Illinois (2015), Indiana, Iowa, Kansas, Kentucky (2014), Louisiana (2014), Maryland, Massachusetts (2015), Minnesota, Missouri (2015), Montana (2014), Nevada, New York, Ohio, Pennsylvania, Texas (2015), Vermont (2014), Virginia, West Virginia (2015), Wisconsin, and Wyoming (2014).

^†^ Percentage is weighted and excludes missing values and responses of don't know, not sure, not asked.

^§^ Estimate suppressed because relative standard error of the estimate was ≥30%.

**TABLE 3:** Prevalence of ever testing and testing in past 12 months for HIV, by gender identity category — Behavioral Risk Factor Surveillance System, 27 states and Guam,* 2014–2015

Gender identity category	Ever tested for HIV		Tested in past 12 months for HIV	
	Adjusted prevalence^¶^ (95% CI)	aPR^¶^ (95% CI)	Adjusted prevalence^¶^ (95% CI)	aPR^¶(^95% CI)
Transgender women	35.6 (29.2–42.6)	0.6 (0.5–0.7)	10.0 (6.5–15.0)	0.5 (0.3–0.7)
Transgender men	31.6 (22.5–42.4)	0.5 (0.4–0.7)	10.2 (5.8–17.5)	0.5 (0.3–0.8)
Cisgender gay and bisexual men^†^	61.8 (59.0–64.6)	Ref	21.6 (19.4–24.0)	Ref
Cisgender heterosexual men and women^§^	35.2 (34.8–35.6)	0.6 (0.5–0.6)	8.6 (8.4–8.9)	0.4 (0.4–0.5)

**Abbreviations:** aPR = adjusted prevalence ratio; CI = confidence interval; HIV = human immunodeficiency virus; Ref = reference category.

* Data were collected in the following jurisdictions: Colorado (2015), Connecticut (2015), Delaware, Georgia (2015), Guam (2014), Hawaii, Idaho, Illinois (2015), Indiana, Iowa, Kansas, Kentucky (2014), Louisiana (2014), Maryland, Massachusetts (2015), Minnesota, Missouri (2015), Montana (2014), Nevada, New York, Ohio, Pennsylvania, Texas (2015), Vermont (2014), Virginia, West Virginia (2015), Wisconsin, and Wyoming (2014).

^†^ Includes cisgender men who reported a sexual orientation of gay or bisexual.

^§^ Includes cisgender men and women who reported a sexual orientation of straight.

^¶^ Adjusted for: race/ethnicity, age, education, annual household income, health insurance, marital status, geographic region, metropolitan county of residence, ever diagnosed with depressive disorder.

## Discussion

Despite the high risk for HIV infection previously reported among transgender populations, nearly two thirds of transgender women and men in the sample reported never testing for HIV, which is consistent with evidence suggesting that many HIV-infected transgender women are not aware of their status (*5*). The prevalences of ever and past year testing among transgender women and men were comparable to those among cisgender heterosexual men and women, a group at much lower risk for infection. Transgender women and men reported a substantially lower prevalence of ever and past year testing than did cisgender gay and bisexual men. These findings indicate that current self-reported HIV testing levels among transgender women and men are inconsistent with their HIV risk profiles. Innovative, tailored approaches might be needed to reach transgender persons who are not being reached by existing HIV prevention strategies that focus on other key populations, such as gay, bisexual, and other men who have sex with men.***

Black transgender women and men were more likely than their white counterparts to report ever testing, which might reflect success of expanded testing measures focused among black communities (*6*) or might be a response to racial/ethnic disparities in HIV infection reported among transgender women (*1*,*5*). Transgender women who ever received a diagnosis of depressive disorder were more likely than those who had not to report ever testing; this is consistent with previous findings in the U.S. general population (*7*). However, few other differences in testing prevalence across demographic subgroups were identified, indicating widespread opportunities for improvement of testing measures aimed toward all transgender women and men who are at risk for HIV infection. Such measures should account for the unique barriers to testing that many transgender persons might face, such as HIV stigma within the transgender community (*8*), gender identity stigma in health care settings (*9*), and socioeconomic marginalization (*10*).

The findings in this report are subject to at least four limitations. First, the proportion of transgender respondents was small (<1%), which reduced the precision of HIV testing estimates. Second, BRFSS transgender data are only representative of transgender persons in the 28 jurisdictions that participated in the optional module and therefore cannot be generalized to the entire U.S. transgender population. Third, the measure of gender identity might incorrectly classify transgender respondents who self-identify simply as man or woman rather than transgender man or woman, which would potentially underestimate the number of transgender persons in the sample. Finally, because BRFSS does not ask questions about HIV status or sexual risk behaviors, the analytic sample might have included respondents who are already living with HIV infection or who are not at risk for HIV infection and therefore would be less likely to have tested for HIV in the past year or at all.

The findings of this analysis indicate suboptimal rates of HIV testing among transgender women and men. The population-based estimates in this report can serve as a baseline for future monitoring of testing trends among transgender persons. Intensified and expanded use of culturally appropriate recruitment methods by public health officials might enhance activities to reach transgender women and men and increase the rates of testing. CDC is currently working to enhance the capacity of community-based organizations to provide targeted HIV testing in addition to other prevention and support services to transgender persons who are at risk for or have newly diagnosed HIV. These programs and other innovative approaches are needed to improve delivery of HIV testing and other prevention services to transgender persons.

**Summary**

**What is already known about this topic?**

Transgender persons are at high risk for HIV infection. CDC recommends that persons at high risk for HIV infection be screened for HIV at least annually, but transgender persons are not specified in the current recommendations, and current nationwide HIV testing rates for transgender persons are unknown.

**What is added by this report?**

This analysis of 2014 and 2015 Behavioral Risk Factor Surveillance System data showed that transgender women and men self-reported a lower prevalence of HIV testing (both ever and in the past year) compared with gay and bisexual men whose gender identities match their sex assignments at birth (cisgender). Transgender women and men self-reported testing at levels similar to cisgender heterosexual men and women.

**What are the implications for public health practice?**

Transgender women and men reported current HIV testing levels that were inconsistent with their HIV risk profiles. Innovative, tailored approaches might be needed to reach transgender persons who are not being reached by existing HIV prevention strategies that focus on other key populations, such as gay, bisexual, and other men who have sex with men.

## Footnotes

* Persons assigned male sex at birth who identify as woman, transgender woman, or another transfeminine identity.

† Persons assigned female sex at birth who identify as man, transgender man, or another transmasculine identity.

§ Persons whose sex assigned at birth is the same as their gender identity or expression.

¶
https://www.cdc.gov/brfss/ .

** Delaware, Guam, Hawaii, Idaho, Indiana, Iowa, Kansas, Kentucky, Louisiana, Maryland, Minnesota, Montana, Nevada, New York, Ohio, Pennsylvania, Vermont, Virginia, Wisconsin, and Wyoming.

†† Colorado, Connecticut, Delaware, Georgia, Hawaii, Idaho, Illinois, Indiana, Iowa, Kansas, Maryland, Massachusetts, Minnesota, Missouri, Nevada, New York, Ohio, Pennsylvania, Texas, Virginia, West Virginia, and Wisconsin.

§§
https://www.cdc.gov/brfss/annual_data/2014/pdf/2014_dqr.pdf .

¶¶
https://www.cdc.gov/brfss/annual_data/2015/pdf/2015-sdqr.pdf .

***
https://effectiveinterventions.cdc.gov/en .
